# Circularly and elliptically polarized light under water and the Umov effect

**DOI:** 10.1038/s41377-019-0143-0

**Published:** 2019-03-20

**Authors:** Yitian Ding, Stanley Pau

**Affiliations:** College of Optical Sciences, University of Arizona, Tucson, AZ 85721 USA

**Keywords:** Optical techniques, Applied optics, Optical techniques, Applied optics

## Abstract

Total internal reflection occurs when light is incident on the interface of high- and low-refractive-index materials at an angle greater than the critical angle. Sunlight with high degree of linear polarization, such as atmospheric scattered skylight, can be converted with a high efficiency up to 53% to circular and elliptical polarizations by total internal reflection under water in the region outside Snell’s window. The degree of circular polarization is observed to be inversely dependent on the albedo of underwater objects and is shown to be a direct consequence of the Umov effect. Our results are important for underwater polarimetry, surveillance applications and studies of marine animals’ polarized vision near the water-air interface.

## Introduction

Circularly polarized (CP) and elliptically polarized (EP) states of light are rarely seen in nature. CP light is observed in radiation from distant celestial bodies^1,2^, reflections from chiral materials^3,4^ and animal skin and exoskeletons^5–7^, bioluminescence^8,9^, and total internal reflection (TIR)^10^ of polarized light. With the exception of astronomical observations and TIR, most CP sources have a biological origin related to enantiomeric excess that is unique and common in life^9,11^. In addition, CP light has been found to be persistent in scattering environments^12,13^, and it can be used in descattering^14^ and reflectometry^15^. The perception of CP and EP light in animal vision has also been studied, and visual systems capable of detecting CP light has been observed in stomatopod crustaceans such as the Mantis shrimp^16^, which utilizes a CP signal as a secure method of communication^7,17^. Crustaceans typically live near shores, where the water is shallow^18^ (5–10 m), i.e., close to the air–water interface.

In this work, we study the visibility of CP and EP light under water for objects of different albedos and show that a high degree of circular polarization (DoCP) can be observed in the region outside Snell’s window^16^, which is caused by a polarization-dependent phase shift from TIR. The well-known Umov effect, which applies to linear polarization (LP) states of light by relating the albedo of an object to the degree of linear polarization (DoLP), has been used for albedo^19^ and particle density estimation^20^, atmospheric measurements in twilight^21^ and the retrieval of the size of near-Earth asteroids^22^. A similar variation in the DoCP is observed when an object is illuminated with polarized light. Our study shows that CP vision permits the discrimination of direct field-of-view and TIR reflections of an object, such as a predator or prey, and this signal is strongly determined by the reflectivity of the object.

The polarization state of polychromatic light is typically described by the Stokes vector ${\mathbf{S}} = (S_0,S_1,S_2,S_3)$. The properties of LP light can be described by the DoLP and the angle of linear polarization (AoLP), while the properties of CP and EP light can be described by the DoCP. The parameters are defined as^23^

1 $$\begin{array}{*{20}{l}} {\mathrm{DoLP}} \hfill & = \hfill & {\frac{{\sqrt {S_1^2 + S_2^2} }}{{S_0^2}}} \hfill \\ {\mathrm{AoLP}} \hfill & = \hfill & {\frac{1}{2}{\kern 1pt} {{\tan}}^{ - 1}{\kern 1pt} \left( {\frac{{S_2}}{{S_1}}} \right)} \hfill \\ {\mathrm{DoCP}} \hfill & = \hfill & {\frac{{S_3}}{{S_0}}} \hfill \end{array}$$

A change in the polarization state through an interaction is described by a Mueller matrix, which is a 4 × 4 transformation matrix of the Stokes vector.

**Fig. 1:**
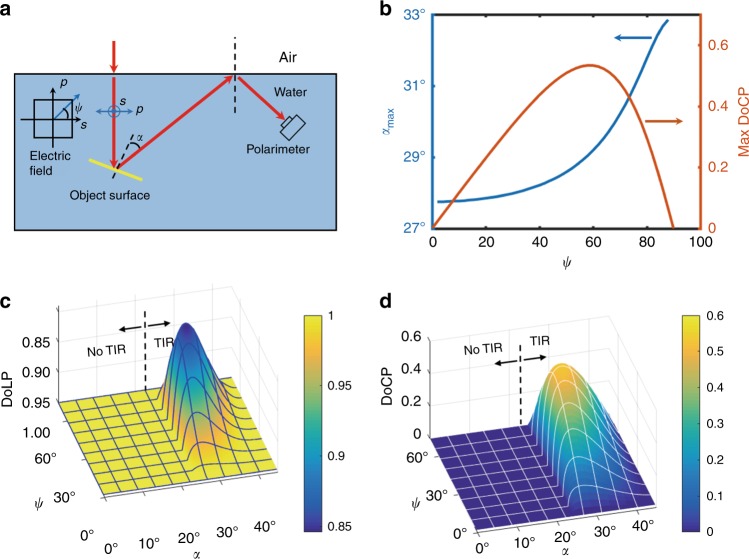
Schematic and analysis of the underwater scene. **a** An illustration of the scene. The incident angle on the surface of the object is *α*. **b** Variation in the maximum DoCP vs. *ψ* (orange) and the maximum DoCP location vs. *ψ* (blue). **c** Variation in the DoLP vs. *α* and *ψ*. Note that the DoLP axis has a reverse direction. **d** Variation in the DoCP vs. *α* and *ψ*

Light from the sky has been observed to be highly polarized with a DoLP up to 0.85 and a predicted DoLP of ~0.94 by single Rayleigh scattering and molecular depolarization^24,25^. The primary mechanisms of polarized light generation are single scattering by gas molecules and small particulates and multiple scatterings by clouds, aerosols, and ground surfaces. In this work, we assume that the water surfaces are illuminated by a linearly polarized beam with a DoLP = 1. The beam, as shown in Fig. 1a, is described by a Stokes vector ${\mathbf{S}} = S_0[1,{{\cos}}{\kern 1pt} \psi ,{{\sin}}{\kern 1pt} \psi ,0]^T$, where *S*_0_ is the irradiance of the beam, *ψ* is the orientation of electric field, and *T* denotes transpose operation. To demonstrate the location of the maximum DoCP and the Umov effect, we assume normal incidence of light on the water surface in this study for simplicity, while a nonzero solar zenith angle or a wavy water surface can result in oblique incidence of light. The oblique incidence leads to a rotation of the plane of LP and a nonsymmetric distribution of the DoCP as a function of the incident angle, and the latter case requires further study. The transmitted beam then reflects from an underwater object, undergoes TIR at the water-air interface, and becomes elliptically or CP. The Mueller matrix for TIR is^26^

2 $${\mathbf{M}}_{\mathrm{TIR}} = \left( {\begin{array}{*{20}{c}} 1 & 0 & 0 & 0 \\ 0 & 1 & 0 & 0 \\ 0 & 0 & {{{\cos}}{\kern 1pt} \delta } & { - {{\sin}}{\kern 1pt} \delta } \\ 0 & 0 & {{{\sin}}{\kern 1pt} \delta } & {{{\cos}}{\kern 1pt} \delta } \end{array}} \right)$$

where $\delta = \delta _s - \delta _p$, $\delta _s = 2{\kern 1pt} {{\tan}}^{ - 1}\left( {\frac{{\sqrt {n^2{\kern 1pt} {{\sin}}^2{\kern 1pt} \theta _i - 1} }}{{n{\kern 1pt} {{\cos}}{\kern 1pt} \theta _i}}} \right)$, $\delta _p = 2{\kern 1pt} {{\tan}}^{ - 1}\left( {\frac{{n\sqrt {n^2{\kern 1pt} {{\sin}}^2{\kern 1pt} \theta _i - 1} }}{{{{\cos}}{\kern 1pt} \theta _i}}} \right)$, *θ*_*i*_ is the incident angle on the water’s surface, and *n* = 1.33 is the refractive index of water. The maximum DoCP detected by a polarimeter and the corresponding orientation of the object surface at the maximum DoCP, *α*_*max*_, are functions of *ψ* (Fig. 1b). The maximum occurs at *α*_*max*_ between 28° and 33°, and it has a peak value of 0.53. Inside Snell’s window, the DoLP remains 1, and no CP light is detected; outside Snell’s window, LP (Fig. 1c) light is converted to EP and CP light (Fig. 1d), with the maximum conversion peaks around *α* = 30°, *ψ* = 60°. Our results show that the TIR of an underwater object illuminated by polarized sky light can have a sizable component of CP or EP light.

**Fig. 2:**
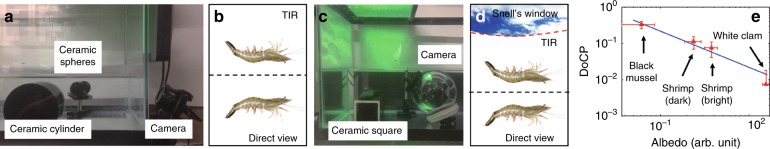
Experimental configurations and corresponding camera views. **a** Configuration with the camera outside the tank. **b** Expected image captured by the camera with the configuration in (**a**). **c** Configuration with the camera enclosed in a water-proof glass globe inside the tank. **d** Expected image captured by the camera with the configuration in (**c**). **e** Inverse relationship between the DoCP and albedo of the four samples under water. Solid line is Eq. (3). Note that the error bars are asymmetric on a log scale. The Stokes images of the shrimp are shown in Supplementary Information

Our calculation is demonstrated by a set of indoor imaging experiments, where a liquid crystal display (LCD) that emits linearly polarized light is used to simulate light from the sky. We consider green light with a wavelength centered at 550 nm, where the LCD emission is peaked. Two configurations are utilized to acquire the Stokes images using an imaging polarimeter placed inside and outside a water tank. The corresponding camera views are illustrated in Fig. 2. The location of the LCD screen can be oriented to reproduce light from the sky at different times of the day.

The Umov effect^19,27^ states that the DoLP of scattered light from an object is inversely proportional to the object’s albedo (*w*). The Supplementary Information contains a rigorous proof of the inverse relationship, which has its origin in unpolarized background scattering, such as subsurface scattering, that is added to a specularly reflected linearly polarized light signal. In this paper, the CP and EP signals detected by the polarimeter are converted from the linearly polarized scattered light through TIR, which has a relatively low loss. A portion of the DoLP in the incident light is converted to the DoCP after TIR with a conversion efficiency independent of the DoLP and a maximum conversion efficiency of 53% (see Supplementary Information). The Umov effect leads to an inverse relationship between the DoCP and the albedo of the object,

3 $${\mathrm{DoCP}} = \frac{{C \cdot \eta (\theta _i,\phi )}}{w}$$

where *C* is a constant, *η* is the conversion efficiency (see Supplementary Information), *θ*_*i*_ is the incident angle on the water-air interface, and *ϕ* is the AoLP of the incident light on the water-air interface. The DoCP of objects with different albedos is shown in Fig. 2(e). The average DoCP and average albedo (*S*_0_) of the four samples under water show an inverse relationship of *DoCP* = 2.2 Wm^−2^/w, where the unit of *w* is Wm^−2^, and DoCP is dimensionless.

**Fig. 3:**
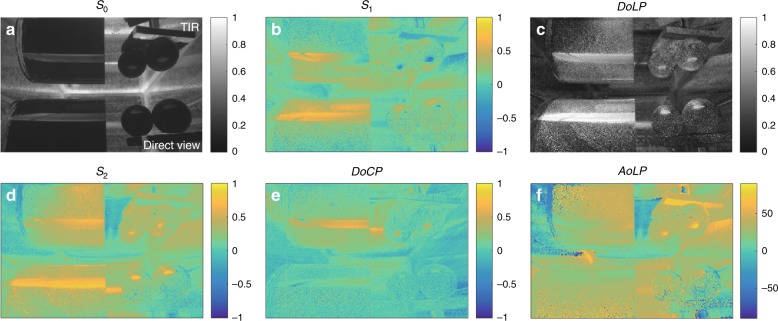
Stokes images of a ceramic cylinder and two ceramic spheres under water

Reflections of low-albedo or dark objects mainly consist of a specular reflection component. Figure 3 shows the direct and TIR views of a black ceramic cylinder and two black ceramic spheres submerged in water. In the direct view, the specular reflection from the objects is linearly polarized with a DoLP ~ 1.0. In the TIR view, the reflected image is EP with a DoLP < 0.9 and a DoCP > 0.5.

**Fig. 4:**
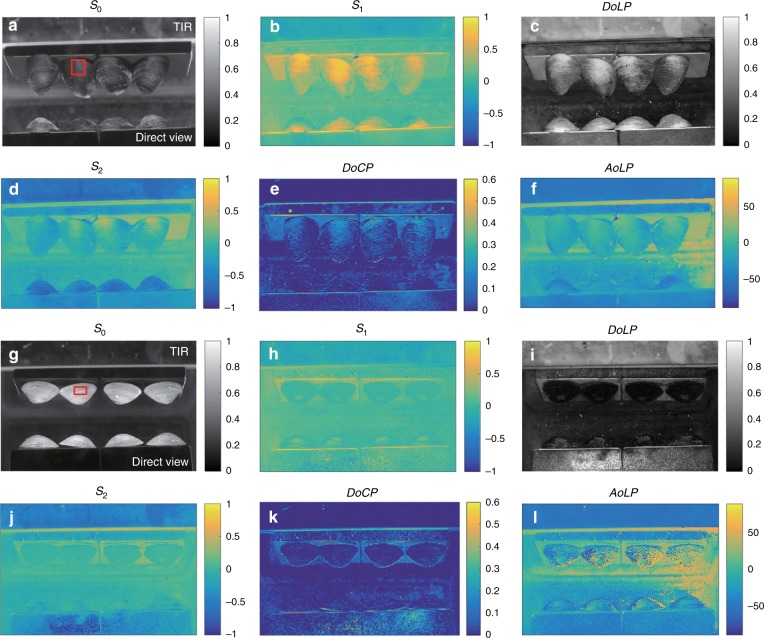
Stokes images of four black mussels and four white clams under water. The regions in red rectangles are used in the study of the Umov effect in Fig. 2(e)

Figure 4a–f shows the direct and TIR views of black mussels under water. The rough exoskeleton of the mussel acts as a depolarizer, which causes multiple scattering of the incident light. In general, both the DoLP and DoCP of the mussels are observed to be less than those of ceramic objects. A high DoLP, up to ~ 1.0, in the direct view, and a high DoCP, up to 0.5, in the TIR view, can be observed.

Figure 4g–l shows the Stokes images of white clams with a high albedo under the same experimental configuration as with the black mussels. A comparison of the clams and mussels shows the effect of the albedo on the polarization state of the reflected light. An inverse relationship between the DoCP and albedo is seen in the TIR view. A high DoCP, up to 0.5, is observed for the black mussels, and a low DoCP, up to 0.1, is observed for the white clams. The Umov effect is seen in the DoLP images. Both the direct and TIR views of the black mussels show a high DoLP, up to 1.0, while those of the white clams show a low DoLP up to 0.2. This is in agreement with the inverse relationship of the Umov effect.

**Fig. 5:**
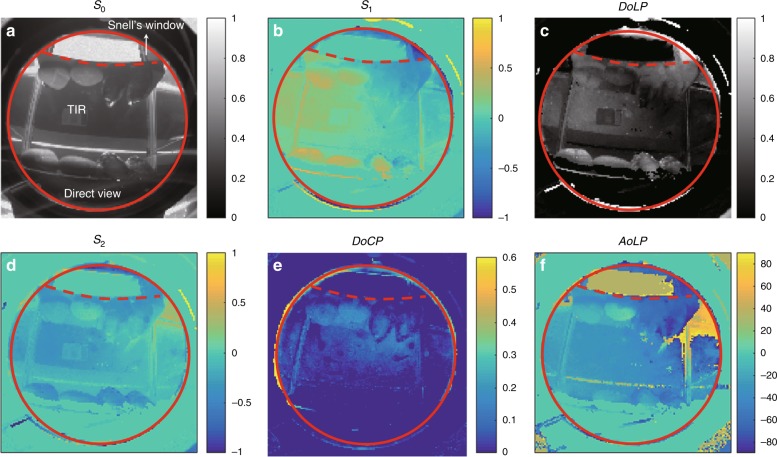
Stokes images of two stones and two black mussels under water. In (**a**-**f**), the region of interest is inside the red solid circle, and Snell’s window is highlighted with a red dashed line. The polarization measurement is noisy outside the region of interest due to the low signal

In the camera-in-water experimental configuration, Snell’s window is captured with both the direct and TIR views as shown in Fig. 5. The object inside Snell’s window is a piece of white printer paper that scatters light and shows a small polarization signal in both views. Meanwhile, a high DoCP is seen in the TIR view, and the DoLP in the direct view is stronger than that in the TIR view. This demonstrates the conversion of the DoLP into DoCP through TIR. The conversion efficiency depends on the incident angle of the light on the water-air interface and the AoLP of the incident light but not the exact value of the DoLP. In general, the DoCP of the TIR light is a linear function of the DoLP of the incident light.

In conclusion, highly CP and EP signals are observed outside Snell’s window for objects under water. There is a large difference in the DoCP and DoLP between the TIR and direct views of underwater objects. For animals with CP vision, these differences can potentially be used to identify a direct view and reflections of predators or prey. Under polarized illumination from the sky, the exact values of the DoCP and DoLP for underwater objects depend on the albedo of the object and can be described by the Umov effect.

## Methods

The polarimeter used in the experiment was constructed with a division of time polarimeter consisting of a Sony *α*6000 camera and three linear, left-hand circular, and right-hand-circular polarizers. The camera’s resolution was 6000 × 4000. The camera’s sensor was covered with a Bayer filter, and only the green color channel was used. The linear polarizer was a HOYA 72 mm PL filter, the left-hand circular polarizer was a B + W F-PRO 72 mm filter, and the right-hand circular polarizer was a HOYA PRO1 72 mm circular PL filter. For various LP measurements at angles of 0°, 45°, 90°, 135°, the linear polarizer was attached in front of the camera lens and rotated manually. The Mueller matrices of the three polarizers were measured with an Axometrics AxoScan polarimeter with an operating range of 400 nm to 900 nm, and the Stokes images of the scene were computed using the polarization data reduction method. Each pixel of the camera was calibrated both radiometrically and polarimetrically using an LCD screen and a power meter.

## Supplementary information

Supplementary Information

## Data Availability

The datasets generated and analyzed during the current study are available from the corresponding author on reasonable request.
