# Supplementary material for: Circularly and elliptically polarized light under water and the Umov effect
Source: Light Sci Appl. 2019 Mar 20;8:32. doi: 10.1038/s41377-019-0143-0 (PMC6425041; doi:10.1038/s41377-019-0143-0)
Supplement: Supplementary file 1 — Supplementary Information [file 41377_2019_143_MOESM1_ESM.docx]

Supplementary: Circularly and elliptically polarized light under water and the Umov effect

Yitian Ding^1^, Stanley Pau^1^

^1^ College of Optical Sciences, University of Arizona, Tucson, Arizona 85721, USA

Correspondence: Yitian Ding ([yding@optics.arizona.edu](mailto:yding@optics.arizona.edu), 585-748-9789) or Stanley Pau ([spau@optics.arizona.edu](mailto:spau@optics.arizona.edu), 520-626-3908)

**Supplementary 1. Stokes images of shrimp with high albedo**


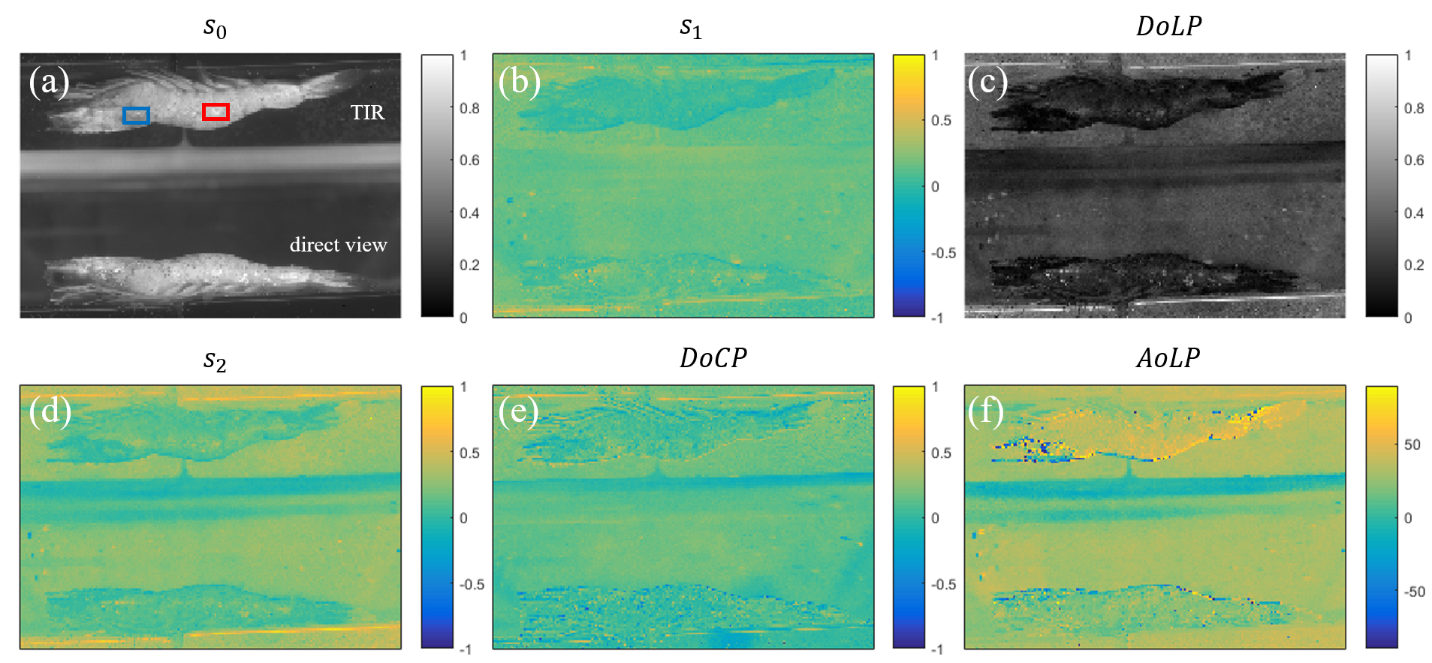


Figure S1. Stokes images of a shrimp under water. Regions in red and blue rectangles are used in the study of the Umov effect in Figure 2(e) in the manuscript. The region in the red rectangle is bright, whereas the region in the blue rectangle is dark.

As shown in Fig. S1, both the direct and TIR views of a shrimp show low polarization signal due to the subsurface scattering in the shrimp’s tissue. The specular component in the reflected light of the shrimp is the direct reflection from its exoskeleton. The diffuse component consists light being transmitted through the exoskeleton, scattered among the shrimp’s tissue via subsurface scattering, and transmitted through the exoskeleton again from the tissue to the water. Compared with the specular component, the diffuse component is stronger due to the high transmittance of the exoskeleton. Since the specular component is polarized and the diffuse component is unpolarized, the bright area on the shrimp shows a low DoLP.

In regions where the shrimps’ tissue absorbs light, the region appears dark, and the diffuse component in the area is less than that of the bright area. Therefore, DoLP in the dark area is higher than in the bright area.

**Supplementary 2. Proof of the inverse relationship between DoLP and albedo in the Umov effect**

The DoLP of scattered light from an object is^1^

$$DoLP=\frac{\Delta p}{p+\left[ H\left( \frac{\mu_{0e}}{K} \right)\cdot H\left( \frac{\mu_{e}}{K} \right)-1 \right]},$$

where $p$ is the volume-average single-particle phase function (VSPF) that describes the angular distribution of scattered light of a group of particles, $\Delta p$ is the difference of VSPF between two orthogonal linear polarization directions (e.g. $0^{\circ}$ and $90^{\circ}$), $H$ is the Ambartsumian-Chandrasekhar H function, $K$ is the porosity coefficient, and $\mu_{0e,e}$ are directional cosines of incident and scattering angles.

An approximated formula of the H function is^1^

$$H\left( x \right)\approx\frac{1+2x}{1+2x\sqrt{1-w}},$$

where $w$ is the volume-average single-scattering albedo. This formula approximates the H function with less than 4% error everywhere. Assuming that $w$ is small compared to 1, the H function can be approximated by the first term in its Taylor expansion at $w=0$, and we have

$$H\left( x \right)\approx\left( 1+2x \right)\cdot\frac{1}{1+2x\left( 1-\frac{1}{2}w \right)}=\left( 1-\frac{x}{1+2x}w \right)^{-1}\approx1+\frac{x}{1+2x}w.$$

Therefore, DoLP can be approximated when $w$ is small as

$$DoLP\approx\frac{\Delta p}{p+\left[ \frac{\mu_{0e}}{K+2\mu_{0e}}+\frac{\mu_{0e}}{K+2\mu_{0e}} \right]w},$$

where the $w^{2}$ term is ignored.

To within 4% of error, $K$ can be approximated as^1^

$$K\approx\left( 1-0.605\cdot\phi^{\frac{2}{3}} \right)^{-1},$$

where $\phi$ is the filling factor. $\phi=0$ when the object is completely occupied by scattering particles, and $\phi=1$ when the object is not occupied. Typically, $\phi\sim0.2-0.6$, and $K\sim1.3-1.8$. For example, Blackbird clay has an average $\phi$ of 0.25^2^ and $K$ of 1.32, and well-sorted sand (sand with similar particle sizes) has $\phi$ = 0.58^3^ and $K$ = 1.73. For the following calculation, we take $K=1.5$ as an example.

When the object is diffuse, the specular reflection is a small portion of the albedo ($p\leq0.1w$). For moderate incident and scattering angles, for example, $45^{\circ}$, DoLP is approximately given by

$$DoLP\approx\frac{\Delta p}{p+w}\approx\frac{\Delta p}{w},$$

which is an inverse relation between the DoLP and the albedo when the signal of specular reflection is fixed.

As low albedo objects and high albedo objects differ mainly in diffuse reflection instead of specular reflection, the two types of objects have similar $\Delta p$, but the high albedo objects have greater $w$; hence, we have the Umov effect

$$DoLP\propto\frac{1}{w}.$$

**Supplementary 3. Derivation of the linear dependence of DoCP of TIR light on DoLP of incident light**

We assume that the incident light on the water-air interface is linearly polarized and has a Stokes vector of $\mathbf{S}=S_{0}\left[ 1,s_{1},s_{2},0 \right]^{T}$, where $S_{0}$ is the irradiance of light, $s_{1,2}$ are two normalized Stokes parameters, and $T$ denotes transpose operation. The DoLP of the incident light is $\sqrt{s_{1}^{2}+s_{2}^{2}}$. The Mueller matrix for TIR, $\mathbf{M}_{TIR}$, is given in Eq. 2 in the manuscript. The Stokes vector of the TIR light is $\mathbf{S}^{'}=\mathbf{M}_{TIR}\cdot\mathbf{S}=S_{0}\left[ 1,s_{1},\cos\delta\cdot s_{2},\sin\delta\cdot s_{2} \right]^{T}$, where $\delta$ is a phase factor defined in the manuscript and depends on the refractive index of water and incident angle $\theta_{i}$. Therefore, the DoCP of TIR light is $\sin\delta\cdot s_{2}$.

The conversion efficiency from linear polarization in the incident light to circular polarization in the TIR light, $\eta$, is defined as the ratio of the energy in the linearly polarized component in the incident light over the energy of the circularly polarized component in the TIR light. The decomposition of the Stokes vector into unpolarized, linearly polarized, and circularly polarized components gives

$$\mathbf{S}=S_{0}\cdot(1-DoLP)\cdot\left( \begin{matrix} 1 \\ 0 \\ 0 \\ 0 \end{matrix} \right)+S_{0}\cdot DoLP\cdot\left( \begin{matrix} 1 \\ s_{1}/DoLP \\ s_{2}/DoLP \\ 0 \end{matrix} \right),$$

and

$$\mathbf{S}^{'}=S_{0}\cdot\left( 1-DoLP^{'}-DoCP \right)\cdot\left( \begin{matrix} 1 \\ 0 \\ 0 \\ 0 \end{matrix} \right)+S_{0}\cdot DoLP'\cdot\left( \begin{matrix} 1 \\ s_{1} \\ \cos\delta\cdot s_{2} \\ 0 \end{matrix} \right)+S_{0}\cdot DoCP\cdot\left( \begin{matrix} 1 \\ 0 \\ 0 \\ 1 \end{matrix} \right),$$

where $DoLP'$ is the DoLP of TIR light. Therefore, the conversion efficiency is

$$\eta=\frac{S_{0}\cdot DoCP}{S_{0}\cdot DoLP}=\frac{\left| \sin\delta\right|\cdot\left| \tan2\phi\right|}{\sqrt{1+\tan^{2} 2\phi}},$$

where $\phi=(1/2)\cdot\tan^{-1} s_{2}/s_{1}$ is the AoLP of incident light. Since neither $\delta$ nor $\phi$ is a function of DoLP, the DoCP of the TIR light is proportional to the DoLP of incident light. If the Umov effect exists for the incident light ($DoLP=C/w$, where $C$ is a constant and $w$ is the albedo of the incident light), an inverse relation is expected for the DoCP of TIR light and the albedo as

$$DoCP=\frac{C\cdot\eta\left( \theta_{i},\phi\right)}{w}.$$

Since $\theta_{i}$ and $\phi$ are independent of each other, the maximum of $\eta(\theta_{i},\phi)$ is the product of the maximum of $\left| \sin\delta(\theta_{i}) \right|$ and the maximum of $f\left( \phi\right)=\left| \tan2\phi\right|/\sqrt{1+\tan^{2} 2\phi}$. As shown in Fig. S2, $f(\phi)$ has a maximum of 1 when $\phi=\pi/4$, and $\left| \sin\delta(\theta_{i}) \right|$ has a maximum of 0.53 when $\theta_{i}=60.1^{\circ}$.


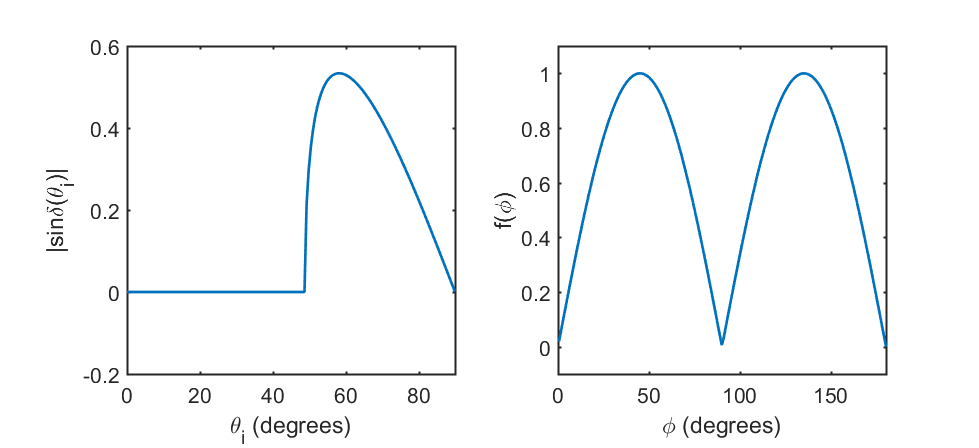


Figure S2. $|\sin\delta(\theta_{i})|$ and $f(\phi)$.

**Supplementary 4. Radiometric calibration of the polarimeter**

A Sony $\alpha$6000 camera was used in the experiment. The image files were saved in TIFF format. The response of each pixel varies across the sensor. To map the pixel values to accurate radiometric values, we performed radiometric calibration of the camera with a Dell U2212MHc LCD screen. The screen showed a white image under a series of ten radiances. The radiance level of the display was controlled by adjusting its Brightness/Contrast tab. The first seven radiance levels were generated by setting the brightness value in the tab equal to 100, 80, 60, 40, 20, and 0, while keeping the contrast value as 100. The other three levels were generated by setting the contrast value to 60, 40, and 20, while keeping the brightness value as 0. The light from the LCD was measured by a Sanwa LP1 laser power meter placed 150 mm to the side of the LCD, with the surface of the sensor $45^{\circ}$ to the normal of the display. Readings of the power meter, $\Phi_{pm}$, were 16.93, 13.84, 11.04, 8.29, 5.53, 2.81, 1.67, 0.69, 0.16 $\mu W$. The radiance density of the LCD, $L_{LCD}^{\lambda}(\lambda)$, is related to $\Phi_{pm}$ as

$$\Phi_{pm}=\int d\lambda\cdot R_{pm}(\lambda)\cdot L_{LCD}^{\lambda}(\lambda)\iint dA_{pm}^{proj}\cdot d\Omega_{pm}$$

where $A_{pm}^{proj}$ and $\Omega_{pm}$ are the projected area and solid angle, respectively, at the power meter, $R_{pm}(\lambda)$, which is the spectral response of the power meter. We assumed that the LCD had a Lambertian emission profile and was uniform. The color filters in LCD typically have narrow spectral bandwidths^4^, and the emission spectrum of an LCD can be approximated by three peaks at the LCD’s red, green, and blue wavelengths, which vary between different LCDs. Therefore, while the color of the LCD remains white, the power meter’s response to the radiances of the red and blue channels of the LCD are proportional to that of the green channel,

$$\int d\lambda\cdot R_{pm}\left( \lambda\right)\cdot L_{LCD}^{\lambda}(\lambda)\approx\left( C_{R}+1+C_{B} \right)\cdot R_{pm}\left( \lambda_{G} \right)\cdot L_{LCD}^{\lambda}\left( \lambda_{G} \right)\cdot\Delta\lambda_{G},$$

where $C_{R,B}$ are the proportional constants for red and blue channels, and $\Delta\lambda_{G}$ is the bandwidth in the approximation of the green channel. Meanwhile, $L_{LCD}^{\lambda}(\lambda)$, is related to the pixel value $\Phi_{cam}(m,n)$, where $m,n$ are the location of pixel on the sensor, as

$$\Phi_{cam}(m,n)=\int d\lambda\cdot R_{cam}(m,n,\lambda)\cdot L_{LCD}^{\lambda}(\lambda)\iint dA_{cam}^{proj}\cdot d\Omega_{cam}$$

where $A_{cam}^{proj}$ and $\Omega_{cam}$ are the projected area and solid angle, respectively, at the camera, $R_{cam}(m,n,\lambda)$, which is the spectral response of the camera pixel at (m, n). We assumed that only the green channel of the camera is used. Note that $\Phi_{cam}(m,n)$ denotes the pixel value and not optical flux, and $R_{cam}(m,n)$ contains the image processes of the camera. Therefore,

$$\Phi_{cam}\left( m,n \right)=C_{geo}\cdot\frac{R_{cam}\left( m,n,\lambda_{G} \right)}{R_{pm}\left( \lambda_{G} \right)\cdot\left( C_{R}+1+C_{B} \right)}\cdot\Phi_{pm},$$

where $C_{geo}$ is a constant that depends only on the geometrical factors of the calibration scene (e.g. distance between LCD and camera). Therefore, the response $R_{cam}\left( m,n,\lambda_{G} \right)$ can be calibrated with $\Phi(m,n)$ and $\Phi_{pm}$.

For a fixed radiance, the camera took 64 images of the screen, and these 64 images were averaged into one image. Then, for each pixel on the average image, the 10 pixel values and 10 corresponding radiances were fit with a spline model, and a lookup table from pixel value to irradiance was obtained. As an example, a hallway scene was studied [Fig. S3(a)]. Pixel responses of 9 sampled points on the image show 27.8% variation in the radiance at pixel value 200 [Fig. S3(b)]. The uncalibrated image [Fig. S3(c)] was brighter than the calibrated image [Fig. S3(d)] due to image processing by the camera, e.g. gamma correction.


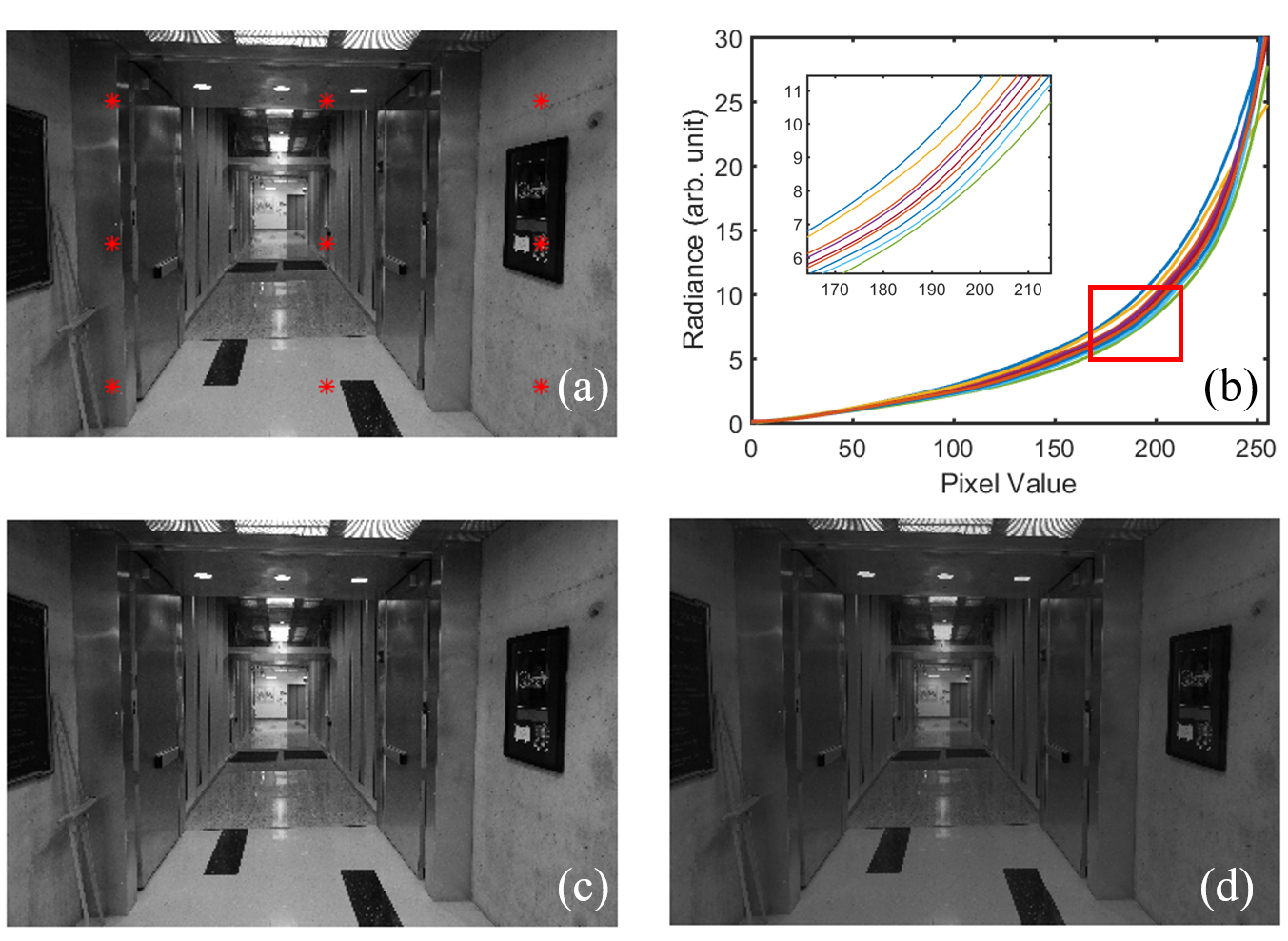


Figure S3. Comparison of calibrated and original images of a hallway scene. (a) Hallway scene images shows nine sample locations denoted as red stars. (b) The radiance value of different pixel is plotted as a function of pixel intensity value. These curves serve as a lookup map for radiometric calibration. The region in red box is shown in the upper left for a better view. (c) The original uncalibrated image. (d) The calibrated image.

**Supplementary 5. Polarimetric calibration of the polarimeter**

Ideal polarizers have an infinitely large extinction ratio, no retardance or depolarization, and their polarization properties are independent of spectrum. Conventional polarizers typically do not completely satisfy all the properties, for example, most circular polarizers for visible spectrum applications are not achromatic and show different retardance for red and blue light. To calibrate for the polarizing filters, the Mueller matrices of the filters, $\mathbf{M}_{LP,RCP,LCP}$, were measured with an AxoScan Mueller matrix polarimeter.

In the experiment, the linear polarizer was used with four orientations, $0^{\circ},45^{\circ},90^{\circ},135^{\circ}$, and the corresponding Mueller matrices are

$$\mathbf{M}_{\gamma}=\mathbf{R}\left( \gamma\right)\cdot\mathbf{M}_{LP}\cdot\mathbf{R}\left( -\gamma\right),$$

where

$$\mathbf{R}\left( \gamma\right)=\left( \begin{matrix} 1 & 0 & 0 & 0 \\ 0 & \cos\gamma& -sin \gamma& 0 \\ 0 & \sin\gamma& \cos\gamma& 0 \\ 0 & 0 & 0 & 1 \end{matrix} \right)$$

is the rotation Mueller matrix, and $\gamma$ is the rotation angle. The analyzer vector of a Mueller matrix, $\mathbf{A}$, is the first row of the matrix, and the polarimetric measurement matrix, $\mathbf{P}$, consists of the analyzer vectors of the Mueller matrices used in one polarimetric measurement. In this experiment,

$$\mathbf{P}=\left( \begin{aligned} \mathbf{A}_{0} \\ \mathbf{A}_{45} \\ \mathbf{A}_{90} \\ \mathbf{A}_{135} \\ \mathbf{A}_{RCP} \\ \mathbf{A}_{LCP} \end{aligned} \right),$$

which is a $6\times4$ matrix. The Stokes vector, $\mathbf{S}_{m,n},$, is related to $\mathbf{P}$ and measured irradiance as

$$\mathbf{I}_{m,n}=\mathbf{P}\cdot\mathbf{S}_{m,n},$$

where $\mathbf{I}_{m,n}$ is a 6-element column vector that consists of the measured irradiances at pixel location $\left( m,n \right)$ with a specific polarizing filter,

$$\mathbf{I}_{m,n}=\left( \begin{aligned} I_{m,n;0} \\ I_{m,n;45} \\ I_{m,n;90} \\ I_{m,n;135} \\ I_{m,n;RCP} \\ I_{m,n;LCP} \end{aligned} \right).$$

Therefore,

$$\mathbf{S}_{m,n}=\mathbf{P}^{+}\cdot\mathbf{I}_{m,n},$$

where $\mathbf{P}^{+}=\left( \mathbf{P}^{T}\cdot\mathbf{P} \right)\cdot\mathbf{P}^{T}$ is the pseudoinverse of $\mathbf{P}$ and $T$ denotes the transpose operation.

In the experiments, eight frames of images were captured and averaged into one image. An estimate of the uncertainties in $S_{0}$ (albedo) is calculated from the variation of captured images in different frames. The variation is caused by sensor and Poisson noises. An estimate of the uncertainties of DoCP is made from multiple measurements of the polarizers’ Mueller matrices. The variation is mainly caused by inaccuracies in the orientation of linear polarizer’s transmission axis.

**References**

1 Hapke, B. *Theory of reflectance and emittance spectroscopy*. (Cambridge: Cambridge University Press, 2012).

2 Shepard, M. K. & Helfenstein, P. A test of the Hapke photometric model. *Journal of Geophysical Research: Planets* **112** (2007).

3 Beard, D. & Weyl, P. Influence of texture on porosity and permeability of unconsolidated sand. *AAPG Bulletin* **57**, 349-369 (1973).

4 Woods, A. J., Yuen, K. L. & Karvinen, K. S. Characterizing crosstalk in anaglyphic stereoscopic images on LCD monitors and plasma displays. *Journal of the Society for Information Display* **15**, 889-898 (2007).
